# Editorial: Application of omics-based technologies and the impact on food science

**DOI:** 10.3389/fmicb.2023.1155757

**Published:** 2023-03-01

**Authors:** Liliana Oliveira Rocha, Nathália Cristina Cirone Silva

**Affiliations:** Department of Food Science and Nutrition, Food Engineering School, University of Campinas, Campinas, Brazil

**Keywords:** metagenomics, transcriptomics, microbiology, microbiome, fermentation

The global demand for food security, sustainability, and international food trade are critical topics in food science and are strictly influenced by food safety. To ensure safe products for consumers, an integrated approach for controlling microbial contamination “from farm to fork” is recommended by government agencies worldwide. In this context, omics-based technologies (e.g., genomics, metagenomics, transcriptomics, proteomics, and metabolomics) have revolutionized the diagnostics of microbial contamination of food, feed, and beverages (King et al., 2017).

The identification of microorganisms that have been introduced throughout the food chain is of utmost importance to certify that food products are free of pathogens and toxins that may cause health issues (Sequino et al., 2022). Utilization of omics-based techniques can give information on known and unknown foodborne pathogens, such as virulence, adaptation, and antimicrobial resistance. The genetic differences among strains can aid in tracking the origin of outbreaks, therefore improving public health systems (Vieira et al., 2022).

This understanding is also important for reducing food waste by monitoring spoilage microorganisms throughout the food chain and product shelf-life; moreover, it may aid in determining the main processing and storage conditions that can affect the occurrence and dynamics of these microorganisms, therefore improving food quality and safety (Sequino et al., 2022).

Additionally, these technologies have shed light on the microbial communities of various food ecosystems, bringing a deeper understanding of their diversity, behavior, metabolism, interactions, and environmental factors involved in their ecology. Metagenomics together with gene expression and metabolome data have allowed the correlation between specific communities and functional predictions in specific environmental conditions. The multi-omics approach has improved the science framework toward synthetic biology, focusing on the utilization of microbial communities for generating valuable products (Ferrocino and Cocolin, 2017).

These observations, together with the knowledge of biological systems, have helped the development of fermented food processes and novel foods with functional attributes, supporting human, and animal health. In this respect, metabolomics combined with other omics technologies is of great interest in identifying biologically active compounds (Nayak et al., 2021). Additionally, studies regarding interactions between intestinal beneficial microorganisms and food compounds have revealed new sources of prebiotics (That et al., 2022); and novel probiotic microorganisms as well as their functional mechanisms have been determined through omics data analyses, and these can be further investigated for developing fermented foods with functional properties (Khullar et al., 2022).

Another important subject is the utilization of omics technologies for recognizing better plant traits and using them for breeding purposes, therefore producing resistant, nutritional and quality food crops (Emon, 2016).

The aim of this Research Topic was to provide updated information on the impact of omics-based technologies on food science and gather significant studies from different areas.

With the advent of high-throughput DNA sequencing-based methods, multiple studies for determining the microbial communities of various food matrices were conducted; however a few reported the issues associated with inaccuracies resulted from DNA of unviable microorganisms. Based on this approach, Yap et al. evaluated 4 different sequencing methods to differentiate viable and non-viable cells in milk. Shotgun metagenomics was performed with and without propidium monoazide (PMA) treatment, a cell viability dye. The authors observed that PMA was adequate for their experiment, but further studies are necessary, especially for applications on more complexes microbiomes.

Using *in silico* metatranscriptomic approach, Almeida et al., studied biofilm-related effectors in metatranscriptomics datasets to understand the mechanisms for bacterial persistence in dairy environments. The authors observed genes involved with flagella, adhesion, and Quorum sensing/Quorum quenching, and demonstrated that the use of metatranscriptomic data is important to understand the regulatory mechanisms in dairy-related biofilms; therefore this tool can be successfully used to improve food safety.

In fermented food, omics technologies have been applied to aid the optimization of processes and the development of better products. Wang et al. observed the production of beneficial metabolites during the co-fermentation of human milk-derived probiotic strains and *Poria cocos*, suggesting an important role of *Poria cocos* as a functional food. Another study, demonstrated that the fermentation of white ginseng (WG) with distinct probiotic strains led to different metabolite composition, which presented different biological activities and enhanced product quality (Chen et al.). Additionally, Shang et al. studied the communities of Daqu and fermented grains for brewing Jiang-flavor Baijiu, a traditional Chinese food, this study was important to screen for indigenous bacteria to be used in this product and to understand the major fermentation steps of the product.

In another context, Montso et al. assessed the caecal microbial communities and their metabolic pathways of broiler chickens after using non-conventional protein sources, such as marama, in the animals' diet. The results revealed that the diet based on marama bean meal could drastically improve caecal microbiota, and can be used to reduce the costs associated with feed and to enhance the broilers' health. Finally, transcriptome analysis was conducted to evaluate the mechanisms involved in the production of docosahexaenoic acid (DHA)-rich lipids by the microalgae *Schizochytrium* sp. The study demonstrated that the use of malate during fed-batch fermentation may enhance DHA production due to the upregulation of genes involved in acetyl-CoA and NADPH algae metabolism.

This topic explored the multifaceted use of omics technologies, aiming to expand the associated knowledge to improve food quality, safety, and the processes involved in the production of functional foods. This collection of manuscripts delivered innovative results and demonstrated some unresolved challenges that need further investigation in food science.

## Author contributions

All authors listed have made equal contributions to the editorial and approved it for publication.
